# Complete convergence of randomly weighted END sequences and its application

**DOI:** 10.1186/s13660-017-1457-1

**Published:** 2017-08-07

**Authors:** Penghua Li, Xiaoqin Li, Kehan Wu

**Affiliations:** 1 Automotive Electronics Engineering Research Center, College of Automation, Chongqing University of Posts and Telecommunications, Chongqing, 400065 China; 2 School of Mathematical Sciences, Anhui University, Hefei, 230601 China; 3 Training Center of Anhui Provincial Electric Power Company, Hefei, 230000 China

**Keywords:** 60E15, 60F15, complete convergence, randomly weighted, END sequences, strong law of large numbers

## Abstract

We investigate the complete convergence of partial sums of randomly weighted extended negatively dependent (END) random variables. Some results of complete moment convergence, complete convergence and the strong law of large numbers for this dependent structure are obtained. As an application, we study the convergence of the state observers of linear-time-invariant systems. Our results extend the corresponding earlier ones.

## Introduction

Let us recall the concept of extended negatively dependent (END) random variables which was introduced by Liu [1].

### Definition 1.1

We call random variables $\{X_{n},n\geq 1\}$ to be END if there exists a positive constant *M* such that both

$$P(X_{i}>{x_{i}}, i=1, 2, \ldots, n)\leq M\prod _{i=1}^{n}P(X_{i}>x_{i}) $$

and

$$P(X_{i}\leq x_{i}, i=1, 2, \ldots, n)\leq M\prod _{i=1}^{n}P(X_{i}\leq x_{i}) $$

hold for each $n\geq1$ and all real numbers $x_{1},x_{2},\ldots,x_{n}$.

Obviously, for all $1\leq i \leq n$, let $x_{i}=-\infty$ or $x_{i}=+\infty$ in Definition 1.1, it is easy to see that the dominating coefficient $M\geq1$. If the dominating coefficient *M* is 1, then END random variables reduce to NOD random variables which contain NA random variables and NSD random variables (see Joag-Dev and Proschan [2], Hu [3] and Wang *et al.* [4]). Various examples of NA random variables and related fields can be found in Bulinski and Shaskin [5], Prakasa Rao [6], Oliveira [7] and the references therein. In view of the importance of END random variables, many researchers pay attention to the study of END. For example, Liu [1, 8] studied the precise large deviations and moderate deviations of END sequence with heavy tails; Chen *et al.* [9] obtained strong law of large numbers of END sequence and gave some applications to the risk theory and renewal theory; Shen [10] obtained some moment inequalities of END sequence; Wang *et al.* [11] and Hu *et al.* [12] investigated the complete convergence for END sequences; Wang *et al.* [13] and Yang *et al.* [14] investigated the nonparametric regression model under END errors; Yang *et al.* [15] obtained some large deviation results of nonlinear regression models under END errors; Wang *et al.* [16] established some exponential inequality for *m*-END sequence; Deng *et al.* [17] studied the Hajek-Renyi-type inequality and strong law of large numbers for END sequences, etc. Furthermore, there are many works on the negatively dependent random variables. For example, Wang *et al.* [18] studied the complete convergence for WOD random variables and gave the application to the estimator of nonparametric regression models; Wu *et al.* [19] obtained some results of complete convergence and complete moment convergence for weighted sums of *m*-NA random variables; Yang and Hu [20] investigated the complete moment convergence of pairwise NQD random variables; Shen *et al.* [21] investigated the strong law of large numbers for NOD random variables; Li *et al.* [22] obtained some results of inverse moment for WOD random variables, etc.

In addition, there are many researchers paying attention to the study of the properties of partial sums of randomly weighted random variables. For example, Thanh and Yin [23] established the complete convergence of partial sums of randomly weighted independent sequences in Banach spaces; Thanh *et al.* [24] investigated complete convergence of partial sums of randomly weighted *ρ̃*-mixing sequences; Cabrera *et al.* [25] and Shen *et al.* [26] investigated the conditional convergence for partial sums of randomly weighted dependent random variables; Yang *et al.* [27] and Yao and Lin [28] obtained the complete convergence and moment of maximum normed based on the randomly weighted martingale differences; Han and Xiang [29] obtained the complete moment convergence of double-indexed randomly weighted sums of *ρ̃*-mixing sequences; Li *et al.* [30] studied the convergence of partial sums of randomly weighted pairwise NQD sequences, etc.

We aim to investigate the complete convergence of partial sums of randomly weighted END sequences. Some results of complete moment convergence, complete convergence and strong law of large numbers for this dependent structure are obtained. As an application, we study the convergence of state observers of linear-time-invariant systems. We extend some results of Thanh *et al.* [24], Wang *et al.* [31] and Yang *et al.* [32] to the case of randomly weighted END sequences. For the details, see our results in Sections 3 and 4, and the conclusions in Section 5. Some lemmas and proofs of main results are presented in Sections 2 and 6, respectively.

## Some lemmas

### Lemma 2.1

Liu [8]

*Let the random variables* $\{X_{n},n\geq1\}$ *be a sequence of END random variables*. *If* $\{f_{n},n\geq1\}$ *is a sequence of all nondecreasing* (*or nonincreasing*) *functions*, *then* $\{f_{n}(X_{n}),n\geq1\}$ *is also a sequence of END random variables*.

### Remark 2.1

Let $\{X_{n},n\geq1\}$ be an END sequence and $\{Y_{n},n\geq1\}$ be a sequence of nonnegative and independent random variables, which is independent of $\{X_{n},n\geq1\}$. Let $Z_{n}=X_{n}Y_{n}$, $n\geq1$. Combining the definition of END with nonnegative and independent of $\{Y_{n}\}$, we establish, for all real numbers $z_{1},\ldots,z_{n}$,

$$\begin{aligned} P(Z_{1}\leq z_{1},\ldots,Z_{n}\leq z_{n}) =&P(X_{1}Y_{1}\leq z_{1}, \ldots,X_{n}Y_{n}\leq z_{n}) \\ =& \int\cdots \int P(X_{1}u_{1}\leq z_{1}, \ldots,X_{n}u_{n}\leq z_{n})\,dF_{Y_{1}}(u_{1}) \cdots dF_{Y_{n}}(u_{n}) \\ \leq& M \int\cdots \int\prod_{i=1}^{n}P(X_{i}u_{i} \leq z_{i})\,dF_{Y_{1}}(u_{1})\cdots dF_{Y_{n}}(u_{n}) \\ =&M\prod_{i=1}^{n}P(X_{i}Y_{i} \leq z_{i}) =M\prod_{i=1}^{n}P(Z_{i} \leq z_{i}), \end{aligned}$$

by using the fact that $u_{1}X_{1},u_{2}X_{2},\ldots,u_{n}X_{n}$ are END random variables following from Lemma 2.1. Similarly, for all real numbers $z_{1},\ldots,z_{n}$, one has

$$\begin{aligned} P(Z_{1}> z_{1},\ldots,Z_{n}>z_{n}) =& \int\cdots \int P(X_{1}u_{1}> z_{1},\ldots ,X_{n}u_{n}>z_{n})\,dF_{Y_{1}}(u_{1}) \cdots dF_{Y_{n}}(u_{n}) \\ \leq& M \int\cdots \int\prod_{i=1}^{n}P(X_{i}u_{i}> z_{i})\,dF_{Y_{1}}(u_{1})\cdots dF_{Y_{n}}(u_{n}) \\ =&M\prod_{i=1}^{n}P(X_{i}Y_{i}> z_{i}) =M\prod_{i=1}^{n}P(Z_{i}>z_{i}). \end{aligned}$$

Therefore, it can be found that $\{Z_{n},n\geq1\}$ is also an END sequence with the same dominating coefficient *M*.

### Lemma 2.2

Shen [10]

*Let* $p\geq2$ *and* $\{X_{n},n\geq 1\}$ *be an END sequence such that* $EX_{n}=0$ *and* $E|X_{n}|^{p}<\infty$ *for all* $n\geq1$. *Then there exists a positive constant* $C_{p}$ *such that for all* $n\geq1$

$$E \Biggl\vert \sum_{i=1}^{n} X_{i} \Biggr\vert ^{p}\leq C_{p}\Biggl\{ \sum _{i=1}^{n} E \vert X_{i} \vert ^{p}+\Biggl(\sum_{i=1}^{n}EX_{i}^{2} \Biggr)^{p/2}\Biggr\} . $$

### Lemma 2.3

Sung [33]

*Let* $\{X_{n},n\geq1\}$ *and* $\{Y_{n},n\geq1\}$ *be sequences of random variables*. *Then*, *for any* $n\geq1$, $q>1$, $\varepsilon>0$ *and* $a>0$,

$$E\Biggl( \Biggl\vert \sum_{i=1}^{n}(X_{i}+Y_{i}) \Biggr\vert -\varepsilon a\Biggr)^{+}\leq \biggl(\frac{1}{\varepsilon^{q}}+ \frac{1}{q-1}\biggr)\frac{1}{a^{q-1}}E \Biggl\vert \sum _{i=1}^{n}X_{i} \Biggr\vert ^{q} +E \Biggl\vert \sum_{i=1}^{n}Y_{i} \Biggr\vert . $$

### Lemma 2.4

Adler and Rosalsky [34] and Adler *et al.* [35]

*Let* $\{X_{n},n\geq1\}$ *be a sequence of random variables which is stochastically dominated by a random variable* *X*, *i*.*e*. $\sup_{n\geq1}P( \vert X_{n} \vert >t)\leq C_{1}P( \vert X \vert >t)$ *for some positive constant* $C_{1}$ *and all* $t\geq0$. *Then*, *for all* $n\geq1$, $\alpha>0$ *and* $\beta>0$, *the following two statements hold*:

$$\begin{aligned}& E\bigl[ \vert X_{n} \vert ^{\alpha}I\bigl( \vert X_{n} \vert \leq \beta\bigr)\bigr] \leq C_{2}\bigl\{ E \bigl[ \vert X \vert ^{\alpha}I\bigl( \vert X \vert \leq\beta\bigr) \bigr]+\beta^{\alpha}P\bigl( \vert X \vert >\beta \bigr)\bigr\} , \\& E\bigl[ \vert X_{n} \vert ^{\alpha}I\bigl( \vert X_{n} \vert >\beta \bigr)\bigr] \leq C_{3}E\bigl[ \vert X \vert ^{\alpha}I\bigl( \vert X \vert >\beta\bigr)\bigr]. \end{aligned}$$

*Consequently*, *one has* $E[ \vert X_{n} \vert ^{\alpha}]\leq C_{4}E \vert X \vert ^{\alpha}$ *for all* $n\geq1$. *Here* $C_{2}, C_{3}, C_{4}$ *are some positive constants not depending on* *n*.

## The complete convergence for partial sums of randomly weighted END sequences

In the following, we list two assumptions:

1. *Let* $\{X_{n},n\geq1\}$ *be a mean zero sequence of END random variables stochastically dominated by a random variables* *X.*
2. *For every* $n\geq1$ *, let* $\{A_{ni},1\leq i\leq n\}$ *be a sequence of independent random variables satisfying that* $\{A_{ni},1\leq i\leq n\}$ *is independent of* $\{X_{n},n\geq1\}$ *.*

### Theorem 3.1

*Assume that* (A.1) *and* (A.2) *are satisfied*. *Let* $\alpha>1/2$, $1< p<2$, $E \vert X \vert ^{p} <\infty$ *and* $\beta\geq1$ *such that*

3.1 $$ \sum_{i=1}^{n}EA_{ni}^{2}=O \bigl(n^{\beta}\bigr). $$

*Then*, *for every* $\varepsilon>0$,

3.2 $$ \sum_{n=1}^{\infty}n^{\alpha p-1-\beta-\alpha}E\Biggl( \Biggl\vert \sum_{i=1}^{n}A_{ni}X_{i} \Biggr\vert -\varepsilon n^{\alpha}\Biggr)^{+}< \infty. $$

*So one has*

3.3 $$ \sum_{n=1}^{\infty}n^{\alpha p-1-\beta}P\Biggl( \Biggl\vert \sum_{i=1}^{n}A_{ni}X_{i} \Biggr\vert > \varepsilon n^{\alpha}\Biggr)< \infty. $$

### Theorem 3.2

*Assume that* (A.1) *and* (A.2) *are satisfied*. *Let* $\alpha>1/2$, $p\geq2$, $E \vert X \vert ^{p} <\infty$ *and* $\beta\geq1$ *such that*

3.4 $$ \sum_{i=1}^{n}E \vert A_{ni} \vert ^{q}=O\bigl(n^{\beta}\bigr) $$

*for some* $q>2(\alpha p-1)/(2\alpha-1)$. *Then we also obtain the results of* (3.2) *and* (3.3).

For some $\beta\geq1$ and $1/2<\alpha<(1+\beta)/2$, we take $\alpha p=1+\beta$ in Theorem 3.2 and establish the following result.

### Theorem 3.3

*Suppose that* (A.1) *and* (A.2) *are fulfilled*. *Let* $\beta\geq1$, $1/2<\alpha<(1+\beta)/2$ *and* $E \vert X \vert ^{(1+\beta)/\alpha} <\infty$. *If*

3.5 $$ \sum_{i=1}^{n}E \vert A_{ni} \vert ^{q}=O\bigl(n^{\beta}\bigr)\quad\textit{for some }q> \frac{2\beta}{2\alpha-1}, $$

*then*, *for every* $\varepsilon>0$,

3.6 $$ \sum_{n=1}^{\infty}n^{-\alpha}E\Biggl( \Biggl\vert \sum_{i=1}^{n}A_{ni}X_{i} \Biggr\vert -\varepsilon n^{\alpha}\Biggr)^{+}< \infty $$

*and*

3.7 $$ \sum_{n=1}^{\infty}P\Biggl( \Biggl\vert \sum _{i=1}^{n}A_{ni}X_{i} \Biggr\vert > \varepsilon n^{\alpha}\Biggr)< \infty. $$

*Thus*, *by the Borel*-*Cantelli lemma and* (3.7), *the strong law of large numbers is as follows*:

3.8 $$ \frac{1}{n^{\alpha}}\sum_{i=1}^{n}A_{ni}X_{i} \rightarrow0,\quad\textit{a.s. as }n\rightarrow\infty. $$

Let $\log x=\ln\max(x,e)$. In addition, for the case $p=1$, we have the following result.

### Theorem 3.4

*Suppose that* (A.1) *and* (A.2) *are fulfilled*. *Let* $\alpha>0$ *and* $E[ \vert X \vert \log \vert X \vert ] <\infty$. *If* (3.1) *holds*, *then*, *for every* $\varepsilon>0$,

3.9 $$ \sum_{n=1}^{\infty}n^{-1-\beta}E\Biggl( \Biggl\vert \sum_{i=1}^{n}A_{ni}X_{i} \Biggr\vert -\varepsilon n^{\alpha}\Biggr)^{+}< \infty. $$

*So one has*

3.10 $$ \sum_{n=1}^{\infty}n^{\alpha-1-\beta}P\Biggl( \Biggl\vert \sum_{i=1}^{n}A_{ni}X_{i} \Biggr\vert >\varepsilon n^{\alpha}\Biggr)< \infty. $$

### Remark 3.1

If $\beta=1$ in (3.1) and (3.4), then the randomly weighted conditions are

3.11 $$ \sum_{i=1}^{n}EA_{ni}^{2}=O(n) $$

and

3.12 $$ \sum_{i=1}^{n}E \vert A_{ni} \vert ^{q}=O(n)\quad\text{for some } q>2(\alpha p-1)/(2 \alpha-1), $$

which are used in Thanh *et al.* [24]. Under the randomly weighted conditions (3.11), (3.12) and other conditions, Thanh *et al.* [24] obtained some complete convergence such as

$$ \sum_{n=1}^{\infty}n^{\alpha p-2}P\Biggl( \max_{1\leq k\leq n} \Biggl\vert \sum_{i=1}^{k}A_{ni}X_{i} \Biggr\vert > \varepsilon n^{\alpha}\Biggr)< \infty $$

for partial sums of randomly weighted *ρ̃*-mixing sequences. Yang *et al.* [32] extended the results of Thanh *et al.* [24] and obtained complete moment convergence such as

$$ \sum_{n=1}^{\infty}n^{\alpha p-2-\alpha}E\Biggl( \max_{1\leq k\leq n} \Biggl\vert \sum_{i=1}^{k}a_{ni}X_{i} \Biggr\vert -\varepsilon n^{\alpha}\Biggr)^{+}< \infty $$

for partial sums of constant weighted martingale differences. In this paper, we weaken the randomly weighted conditions such as (3.1) and (3.4) for $\beta\geq1$ and obtain the results of Theorems 3.1-3.4. Generally, we extend the results of Thanh *et al.* [24] and Yang *et al.* [32] to the case of randomly weighted END sequences.

## The application to linear-time-invariant systems

As an application of Theorem 3.3, we study the convergence of the state observers of linear-time-invariant systems in this section.

For $t\ge0$, we consider a multi-input-single-output (MISO) linear time invariant system as follows:

4.1 $$ \textstyle\begin{cases} \dot{x}(t) =Ax(t)+Bu(t), \\ y(t)=Cx(t), \end{cases} $$

where $A\in R^{m_{0}\times m_{0}}$, $B\in R^{m_{0}\times m_{1}}$, $C\in R^{1\times m_{0}}$ are known system matrices, $u(t)\in R^{m_{1}}$ is the control input, $x(t)\in R^{m_{0}}$ is the state and $y(t)\in R$ is the system output. The initial state $x(0)$ is unknown. For some limited observations on $y(t)$, it is interesting to estimate $x(t)$. In the setup, the output $y(t)$ is only measured at a sequence of sampling time instants $\{t_{i}\}$ with measured values $\gamma (t_{i})$ and noised $d_{i}$ such that

$$ y(t_{i})=\gamma(t_{i})+d_{i},\quad1\leq i\leq n. $$

We would like to estimate the state $x(t)$ from information on $u(t)$, $\{t_{i}\}$ and $\{\gamma(t_{i})\}$. Let $G^{\prime}$ denote the transpose of *G*. In order to proceed, we need the following assumption.

### Assumption 4.1

The system (4.1) is observable, *i.e.*, the observability matrix

$$W_{o}^{\prime}=\bigl[C^{\prime},(CA)^{\prime}, \ldots,\bigl(CA^{m_{0}-1}\bigr)^{\prime}\bigr] $$

has full rank.

The solution to system (4.1) can be checked:

$$ x(t)=e^{A(t-t_{0})}x(t_{0})+ \int_{t_{0}}^{t}e^{A(t-\tau)}Bu(\tau)\,d\tau. $$

From $\{t_{i},1\leq i\leq n\}$, it follows that

$$ \gamma(t_{i})+d_{i}=y(t_{i})=Ce^{A(t_{i}-t_{n})}x(t_{n})+C \int _{t_{n}}^{t_{i}}e^{A(t_{i}-\tau)}Bu(\tau)\,d\tau. $$

Denote

$$v(t_{i},t_{n})=C \int_{t_{n}}^{t_{i}}e^{A(t_{i}-\tau)}Bu(\tau)\,d\tau. $$

So this leads to the observation

4.2 $$ Ce^{A(t_{i}-t_{n})}x(t_{n})=\gamma(t_{i})-v(t_{i},t_{n})+d_{i}, \quad1\leq i\leq n. $$

Define

$$\begin{aligned}& \Phi_{n}= \begin{bmatrix} Ce^{A(t_{1}-t_{n})} \\ \vdots\\ Ce^{A(t_{n-1}-t_{n})} \\ C \end{bmatrix} ,\qquad \Gamma_{n}= \begin{bmatrix} \gamma(t_{1}) \\ \vdots \\ \gamma(t_{n-1}) \\ \gamma(t_{n}) \end{bmatrix} ,\qquad V_{n}= \begin{bmatrix} v(t_{1},t_{n}) \\ \vdots \\ v(t_{n-1},t_{n}) \\ 0 \end{bmatrix} ,\qquad D_{n}= \begin{bmatrix} d_{1} \\ \vdots \\ d_{n-1} \\ d_{n} \end{bmatrix} . \end{aligned}$$

Then we rewrite (4.2) as follows:

4.3 $$ \Phi_{n}x(t_{n})=\Gamma_{n}-V_{n}+D_{n}. $$

Suppose that $\Phi_{n}$ is full rank, which will be established later. Then the least-squares estimator of $x(t_{n})$ is given by

4.4 $$ \hat{x}(t_{n})=\bigl(\Phi_{n}^{\prime} \Phi_{n}\bigr)^{-1}\Phi_{n}^{\prime}(\Gamma _{n}-V_{n}). $$

Combining (4.3) with (4.4), the estimation error for $x(t_{n})$ at $t_{n}$ is presented as

$$ e(t_{n})=\hat{x}(t_{n})-x(t_{n})=\bigl( \Phi_{n}^{\prime}\Phi_{n}\bigr)^{-1} \Phi_{n}^{\prime}D_{n} =\biggl(\frac{1}{n^{r}} \Phi_{n}^{\prime}\Phi_{n}\biggr)^{-1} \frac{1}{n^{r}}\Phi_{n}^{\prime }D_{n}\quad\text{for some } \frac{1}{2}< r< 1. $$

In order to obtain the convergence, one must consider a typical entry in $\frac{1}{n^{r}}\Phi_{n}^{\prime}D_{n}$. By the Cayley-Hamilton theorem [36], the matrix exponential can be expressed by a polynomial function of *A* of order at most $m_{0}-1$:

$$ e^{At}=\alpha_{1}(t)I+\cdots+\alpha_{m_{0}}(t)A^{m_{0}-1}, $$

where the time functions $\alpha_{i}(t)$ can be derived by the Lagrange-Hermite interpolation method [36]. So one has

$$ Ce^{A(t_{i}-t_{n})}=\bigl[\alpha_{1}(t_{i}-t_{n}), \ldots,\alpha_{m_{0}}(t_{i}-t_{n})\bigr] \begin{bmatrix} C \\ CA \\ \vdots \\ CA^{m_{0}-1} \end{bmatrix} =\varphi^{\prime}(t_{i}-t_{n})W_{o}, $$

where $\varphi^{\prime}(t_{i}-t_{n})=[\alpha_{1}(t_{i}-t_{n}),\ldots,\alpha_{m_{0}}(t_{i}-t_{n})]$ and $W_{o}$ is the observability matrix.

Denote

$$ \Psi_{n}= \begin{bmatrix} \varphi^{\prime}(t_{1}-t_{n}) \\ \vdots \\ \varphi^{\prime}(0) \end{bmatrix} . $$

Then

$$ \Phi_{n}=\Psi_{n}W_{o}, $$

which reduces to

$$\begin{aligned}& \frac{1}{n^{r}}\Phi_{n}^{\prime}\Phi_{n}=W_{o}^{\prime} \frac{1}{n^{r}}\Psi _{n}^{\prime}\Psi_{n}W_{o}, \\& \frac{1}{n^{r}}\Phi_{n}^{\prime}D_{n}= \frac{1}{n^{r}}W_{o}^{\prime}\Psi _{n}^{\prime}D_{n}. \end{aligned}$$

As a result, one has for any $r>0$

4.5 $$ e(t_{n})=\biggl(\frac{1}{n^{r}}\Phi_{n}^{\prime} \Phi_{n}\biggr)^{-1}\frac{1}{n^{r}}\Phi _{n}^{\prime}D_{n} =W_{o}^{-1} \biggl(\frac{1}{n^{r}}\Psi_{n}^{\prime}\Psi_{n} \biggr)^{-1}\frac{1}{n^{r}}\Psi _{n}^{\prime}D_{n}. $$

By Assumption 4.1, it can be found that $W_{0}^{-1}$ exists. The convergence analysis will be established by the sufficiently conditions: $\frac{1}{n^{r}}\Psi_{n}^{\prime}D_{n}\rightarrow0$, a.s., and $\frac{1}{n^{r}}\Psi_{n}^{\prime}\Psi_{n}\geq{\lambda} I$, a.s., for some $\lambda>0$. So we need the following PE condition which was used in Thanh *et al.* [24] and Wang *et al.* [31].

### Assumption 4.2

For some $1/2< r<1$,

4.6 $$ {\lambda}=\inf_{n\geq1}\sigma_{\min} \biggl( \frac{1}{n^{r}}\Psi_{n}^{\prime}\Psi_{n}\biggr)>0,\quad \mbox{a.s.}, $$

where $\sigma_{\min}(H)$ is the small eigenvalue of *H* for a suitable symmetric *H*.

We focus on the convergence of partial sums of randomly weighted END random variables such that

4.7 $$ \frac{1}{n^{r}}\sum_{i=1}^{n} A_{ni}d_{i} $$

for some $1/2< r<1$. Since a typical entry of $\frac{1}{n^{r}}\Psi_{n}^{\prime}D_{n}$ is

4.8 $$ \frac{1}{n^{r}}\sum_{i=1}^{n} \alpha_{j}(t_{i}-t_{n})d_{i}, $$

the convergence analysis for $e(t_{n})$ is a special case of (4.7). Note that when the sampling time sequence is a random process, so are $\alpha_{j}(t_{i}-t_{n})$ in (4.8), rendering a randomly weighted noise driven by END random variables. As an application of Theorem 3.3, we obtain the following theorem.

### Theorem 4.1

*Let* $\beta\geq1$, $1/2< r<1$ *and Assumptions* 4.1 *and* 4.2 *hold*. *Suppose that* $\{d_{n},n\geq1\}$ *is an END sequences stochastically dominated by a random variable* *d* *with* $E \vert d \vert ^{(1+\beta)/r}<\infty$. *Suppose that*, *for some* $q>\frac{2\beta}{2r-1}$, *one has*

4.9 $$ \sum_{i=1}^{n}E \bigl\vert \alpha_{j}(t_{i}-t_{n}) \bigr\vert ^{q}=O\bigl(n^{\beta}\bigr), $$

*where* $1\leq j\leq m_{0}$. *Then*

4.10 $$ \frac{1}{n^{r}} \bigl\Vert \Psi_{n}^{\prime}D_{n} \bigr\Vert \rightarrow 0,\quad \textit{a.s.} $$

*Consequently*,

4.11 $$ e(t_{n})\rightarrow0,\quad \textit{a.s.} $$

### Remark 4.1

If $\{\varphi(t_{i}-t_{n})\}$ is uniformly bounded, a.s., then the condition (4.9) holds with $\beta=1$ for any *q*. Wang *et al.* [31] obtained (4.11) for constant weighted *ρ̃*-mixing errors (see Theorem 4 of Wang *et al.* [31]). Thanh *et al.* [24] extended the result of Wang *et al.* [31] to randomly weighted *ρ̃*-mixing errors (see Theorem 4.1 of Thanh *et al.* [24]). Yang *et al.* [32] obtained the result (4.11) for the case of constant weighted martingale differences (see Theorem 11 of Yang *et al.* [32]). Generally, our Theorem 4.1 generalize the results of Thanh *et al.* [24], Wang *et al.* [31] and Yang *et al.* [32] to the case of randomly weighted END errors.

## Conclusions

In this paper, we investigate the complete convergence of partial sums of randomly weighted END random variables. Some results of complete moment convergence, complete convergence and strong law of large numbers for this dependent structure are presented (see our Theorems 3.1-3.4). As an application of Theorem 3.3, we study the convergence of the state observers of linear-time-invariant systems and obtain the result of the strong law of large numbers for the systems (see our Theorem 4.1). Therefore, we extend some results of Thanh *et al.* [24], Wang *et al.* [31] and Yang *et al.* [32] to the case of randomly weighted END sequences. Furthermore, END random variables contain NA random variables, NOD random variables and NSD random variables, so the results obtained in this paper hold true for these negatively dependent random variables.

## The proofs of main results

In the proofs, $C, C_{1}, C_{2},\ldots$ denote some positive constants not depending on *n*.

### Proof of Theorem 3.1

Since $A_{ni}X_{i}=A_{ni}^{+}X_{i}-A_{ni}^{-}X_{i}$, without loss of generality, we assume $A_{ni}\geq0$ in the proof. For $n\geq1$ and $1\leq i \leq n$, let

$$\begin{aligned}& X_{ni}=-n^{\alpha}I\bigl(X_{i}< -n^{\alpha} \bigr)+X_{i}I\bigl( \vert X_{i} \vert \leq n^{\alpha}\bigr)+n^{\alpha}I\bigl(X_{i}>{n^{\alpha}} \bigr), \\& \tilde{X}_{ni}=n^{\alpha}I\bigl(X_{i}< -n^{\alpha} \bigr)+X_{i}I\bigl( \vert X_{i} \vert >n^{\alpha} \bigr)-n^{\alpha}I\bigl(X_{i}>{n^{\alpha}}\bigr). \end{aligned}$$

It can be found that

$$A_{ni}X_{i}=\bigl[A_{ni}X_{ni}-E(A_{ni}X_{ni}) \bigr]+E(A_{ni}X_{ni})+A_{ni}\tilde {X}_{ni},\quad 1\leq i\leq n. $$

Therefore, by Lemma 2.3 with $a=n^{\alpha}$ and $q=2$, we obtain

6.1 $$\begin{aligned}& \sum_{n=1}^{\infty}n^{\alpha p-1-\beta-\alpha}E\Biggl( \Biggl\vert \sum_{i=1}^{n} A_{ni}X_{i} \Biggr\vert -\varepsilon n^{\alpha} \Biggr)^{+} \\& \quad \leq C_{1}\sum_{n=1}^{\infty}n^{\alpha p-1-\beta-2\alpha}E\Biggl\vert \sum_{i=1}^{n} \bigl[A_{ni}X_{ni}-E(A_{ni}X_{ni})\bigr] \Biggr\vert ^{2} \\& {}\qquad +\sum_{n=1}^{\infty}n^{\alpha p-1-\beta-\alpha}E \Biggl\vert \sum_{i=1}^{n}A_{ni} \tilde{X}_{ni} \Biggr\vert +\sum_{n=1}^{\infty}n^{\alpha p-1-\beta-\alpha} \Biggl\vert \sum_{i=1}^{n}E(A_{ni}X_{ni}) \Biggr\vert \\& \quad :=H_{1}+H_{2}+H_{3}. \end{aligned}$$

Combining (3.1) with Hölder’s inequality, one has

6.2 $$ \sum_{i=1}^{n} E \vert A_{ni} \vert \leq\Biggl(\sum_{i=1}^{n} EA^{2}_{ni}\Biggr)^{1/2}\Biggl(\sum _{i=1}^{n} 1\Biggr)^{1/2}\leq C_{1}n^{\frac{\beta+1}{2}}\leq C_{1}n^{\beta}, $$

by using the fact $\beta\geq1$.

Since, for every $n\geq1$, $\{A_{ni},1\leq i\leq n\}$ is independent of the sequence $\{X_{n},n\geq1\}$, one has by Markov’s inequality, Lemma 2.4, (6.2) and $E \vert X \vert ^{p}<\infty$ ($p>1$)

6.3 $$\begin{aligned} H_{2} \leq&3\sum_{n=1}^{\infty}n^{\alpha p-1-\beta-\alpha}\sum_{i=1}^{n}E \vert A_{ni} \vert E \vert X_{i} \vert I\bigl( \vert X_{i} \vert >n^{\alpha}\bigr) \\ \leq& C_{1}\sum_{n=1}^{\infty}n^{\alpha p-1-\alpha}E\bigl[ \vert X \vert \vert I\bigl( \vert X \vert>n^{\alpha}\bigr)\bigr] \\ =&C_{1}\sum_{n=1}^{\infty}n^{\alpha p-1-\alpha}\sum_{m=n}^{\infty}E\bigl[ \vert X \vert \vert I\bigl(m< \vert X\vert^{1/\alpha}\leq m+1\bigr) \bigr] \\ =&C_{1}\sum_{m=1}^{\infty}E\bigl[ \vert X \vert \vert I\bigl(m< \vert X\vert^{1/\alpha}\leq m+1\bigr) \bigr]\sum_{n=1}^{m}n^{\alpha(p-1)-1} \\ \leq&C_{2}\sum_{m=1}^{\infty}m^{\alpha p-\alpha}E \vert X \vert I\bigl(m< \vert X \vert ^{1/\alpha} \leq m+1\bigr)]\leq C_{3}E \vert X \vert ^{p}< \infty. \end{aligned}$$

In addition, it can be seen that $E(A_{ni}X_{i})=EA_{ni}EX_{i}=0$, $1\leq i\leq n$, $n\geq1$. So, by the proof of (6.3), we have

6.4 $$\begin{aligned} H_{3} =&\sum_{n=1}^{\infty}n^{\alpha p-1-\beta-\alpha}\Biggl\vert \sum_{i=1}^{n} \bigl[-n^{\alpha}EA_{ni}I\bigl(X_{i} < -n^{\alpha}\bigr)-EA_{ni}X_{i}I\bigl( \vert X_{i} \vert > n^{\alpha}\bigr)\cdots \\ &{}+\cdots n^{\alpha}EA_{ni}X_{i}I\bigl( \vert X_{i} \vert >n^{\alpha }\bigr)\bigr]\Biggr\vert \\ \leq&C_{1}\sum_{n=1}^{\infty}n^{\alpha p-1-\beta-\alpha}\sum_{i=1}^{n}E \vert A_{ni} \vert E\bigl[ \vert X_{i} \vert I\bigl( \vert X_{i} \vert >n^{\alpha}\bigr)\bigr]\leq C_{2}E \vert X \vert ^{p}< \infty. \end{aligned}$$

In view of Lemma 2.1, one sees that $\{X_{ni},1\leq i\leq n\} $ are END random variables. Combining the assumption of $\{A_{ni}\}$ with Remark 2.1, we establish that $\{[A_{ni}X_{ni}-E(A_{ni}X_{ni})],1\leq i\leq n\}$ are mean zero END random variables with the same dominating coefficient. So, by Markov’s inequality, (3.1), Lemma 2.2 with $p=2$ and Lemma 2.4, we get

6.5 $$\begin{aligned} H_{1} =&C_{1}\sum_{n=1}^{\infty}n^{\alpha p-1-\beta-2\alpha}E\Biggl\vert \sum_{i=1}^{n} \bigl[A_{ni}X_{ni}-E(A_{ni}X_{ni})\bigr] \Biggr\vert ^{2} \\ \leq&C_{2}\sum_{n=1}^{\infty}n^{\alpha p-1-\beta-2\alpha}\sum_{i=1}^{n}E(A_{ni}X_{ni})^{2} \\ \leq&C_{3}\sum_{n=1}^{\infty}n^{\alpha p-1-2\alpha}E\bigl[X^{2}I\bigl( \vert X \vert \leq n^{\alpha}\bigr)\bigr]+C_{4}\sum_{n=1}^{\infty}n^{\alpha p-1} EI\bigl( \vert X \vert >n^{\alpha}\bigr) \\ :=&C_{3}H_{11}+C_{4}H_{12}. \end{aligned}$$

Since $p<2$ and $EX^{p}<\infty$, it can be checked that

6.6 $$\begin{aligned} H_{11} =&\sum_{n=1}^{\infty}n^{\alpha p-1-2\alpha}\sum_{i=1}^{n} E \bigl[X^{2}I\bigl((i-1)^{\alpha}< \vert X \vert \leq i^{\alpha }\bigr)\bigr] \\ =&\sum_{i=1}^{\infty}E\bigl[X^{2}I \bigl((i-1)^{\alpha}< \vert X \vert \leq i^{\alpha}\bigr)\bigr] \sum_{n=i}^{\infty}n^{\alpha p-1-2\alpha} \\ \leq&C_{1}\sum_{i=1}^{\infty}E \vert X \vert ^{p}X^{2-p}I\bigl((i-1)^{\alpha}< \vert X \vert \leq i^{\alpha}\bigr)]i^{\alpha p-2\alpha}\leq C_{1}E \vert X \vert ^{p}< \infty. \end{aligned}$$

By the proof of (6.3), it follows that

6.7 $$ H_{12}\leq\sum_{n=1}^{\infty}n^{\alpha p-1-\alpha}E\bigl[ \vert X \vert I\bigl( \vert X \vert >n^{\alpha}\bigr)\bigr]\leq CE \vert X \vert ^{p}< \infty. $$

Combining (6.1) with (6.3)-(6.7), we can get (3.2) immediately. Moreover, by (3.2) and Remark 2.6 of Sung [33], for every $\varepsilon>0$, it can be argued that

6.8 $$\begin{aligned} \infty >&\sum_{n=1}^{\infty}n^{\alpha p-1-\beta-\alpha}E \Biggl({ \Biggl\vert \sum_{i=1}^{n}A_{ni}X_{i} \Biggr\vert }-\varepsilon n^{\alpha}\Biggr)^{+} \\ \geq&\sum_{n=1}^{\infty}n^{\alpha p-1-\beta} \int_{0}^{\varepsilon n^{\alpha}} P\Biggl({ \Biggl\vert \sum _{i=1}^{n}A_{ni}X_{i} \Biggr\vert }-\varepsilon n^{\alpha }>t\Biggr)\,dt \\ \geq& \varepsilon\sum_{n=1}^{\infty}n^{\alpha p-1-\beta}P\Biggl({ \Biggl\vert \sum_{i=1}^{n}A_{ni}X_{i} \Biggr\vert }>2\varepsilon n^{\alpha}\Biggr), \end{aligned}$$

which implies (3.3). □

### Proof of Theorem 3.2

We use the same notation in the proof of Theorem 3.1. Obviously, by $p\geq2$, it is easy to see that $q>2(\alpha p-1)/(2\alpha-1)\geq2$. Consequently, for any $1\leq r\leq2$, by Hölder’s inequality and condition (3.4), one has

6.9 $$ \sum_{i=1}^{n} E \vert A_{ni} \vert ^{r}\leq\Biggl(\sum_{i=1}^{n} E \vert A_{ni} \vert ^{q}\Biggr)^{r/q}\Biggl( \sum_{i=1}^{n} 1\Biggr)^{1-r/q}\leq C_{1}n^{\beta\frac{r}{q}+1-\frac{r}{q}}. $$

It can be seen that $\beta\frac{r}{q}+1-\frac{r}{q}-\beta=(\beta -1)(\frac{r}{q}-1){\leq0}$. So one has $\sum_{i=1}^{n} E \vert A_{ni} \vert =O(n^{\beta})$. Together with (6.1), (6.3), (6.4) and (6.9), we obtain $H_{2}<\infty$ and $H_{3}<\infty$. Therefore, we have to prove that $H_{1}<\infty$. Since $q>2$, similar to the proof of (6.5), by Lemma 2.2, it follows that

6.10 $$ \begin{aligned}[b] H_{1}&=C_{1}\sum_{n=1}^{\infty}n^{\alpha p-1-\beta-q\alpha}E\Biggl\vert \sum_{i=1}^{n} \bigl[A_{ni}X_{ni}-E(A_{ni}X_{ni})\bigr] \Biggr\vert ^{q} \\ &\leq C_{2}\sum_{n=1}^{\infty}n^{\alpha p-1-\beta-q\alpha}\Biggl(\sum_{i=1}^{n}E \bigl[A_{ni}X_{ni}-E(A_{ni}X_{ni}) \bigr]^{2}\Biggr)^{q/2} \\ &\quad {}+C_{2}\sum_{n=1}^{\infty}n^{\alpha p-1-\beta-q\alpha}\sum_{i=1}^{n}E \bigl\vert A_{ni}X_{ni}-E(A_{ni}X_{ni}) \bigr\vert ^{q} \\ &:=C_{2}H_{11}+C_{2}H_{12}. \end{aligned} $$

Obviously, for $1\leq i\leq n$, by Lemma 2.4, it follows that

6.11 $$ \begin{aligned}[b] &E\bigl[A_{ni}X_{ni}-E(A_{ni}X_{ni}) \bigr]^{2}\\ &\quad \leq CEA^{2}_{ni}EX_{ni}^{2} \\ &\quad \leq CEA^{2}_{ni}\bigl\{ E\bigl[X^{2}I\bigl( \vert X \vert \leq n^{\alpha}\bigr)\bigr]+n^{2\alpha}P\bigl( \vert X \vert >n^{\alpha}\bigr)\bigr\} \\ &\quad \leq CEA^{2}_{ni}\bigl\{ E\bigl[X^{2}I\bigl( \vert X \vert \leq n^{\alpha}\bigr)\bigr]+E\bigl[X^{2}I\bigl( \vert X \vert >n^{\alpha}\bigr)\bigr]\bigr\} = CEA^{2}_{ni}EX^{2}. \end{aligned} $$

By $p\geq2$ and $E \vert X \vert ^{p}<\infty$, one concludes that $EX^{2}<\infty$. Thus, we take (6.9) with $r=2$ and (6.11), and establish

6.12 $$\begin{aligned} H_{11} \leq& C_{3}\sum_{n=1}^{\infty}n^{\alpha p-1-\beta-q\alpha}\Biggl(\sum_{i=1}^{n}EA^{2}_{ni}EX^{2} \Biggr)^{q/2} \\ \leq& C_{4}\sum_{n=1}^{\infty}n^{\alpha p-1-\beta-q\alpha+(2\beta /q+1-2/q)q/2} \\ =&{C_{4}\sum_{n=1}^{\infty}n^{\alpha p-q\alpha+q/2-2}< \infty}, \end{aligned}$$

by the fact $q>2(\alpha p-1)/(2\alpha-1)$. In addition, by the $C_{r}$ inequality, Lemma 2.4 and (3.4),

6.13 $$\begin{aligned} H_{12} \leq& C_{5}\sum_{n=1}^{\infty}n^{\alpha p-1-\beta-q\alpha}\sum_{i=1}^{n}E \vert A_{ni} \vert ^{q}E \vert X_{ni} \vert ^{q} \\ \leq& C_{6}\sum_{n=1}^{\infty}n^{\alpha p-1-q\alpha}E\bigl[ \vert X \vert ^{q}I\bigl( \vert X \vert \leq n^{\alpha}\bigr)\bigr]+C_{7}\sum _{n=1}^{\infty}n^{\alpha p-1}P\bigl( \vert X \vert >n^{\alpha}\bigr) \\ :=&C_{6}H^{*}_{12}+C_{7}H^{**}_{12}. \end{aligned}$$

By $p\geq2$ and $\alpha>1/2$, one has $2(\alpha p-1)/(2\alpha-1)-p\geq0$, which implies $q>p$. So, by $E \vert X \vert ^{p}<\infty$, it can be argued that

6.14 $$\begin{aligned} H^{*}_{12} =&\sum_{n=1}^{\infty}n^{\alpha p-1-q\alpha}\sum_{i=1}^{n} E\bigl[ \vert X \vert ^{q}I\bigl((i-1)^{\alpha}< \vert X \vert \leq i^{\alpha}\bigr)\bigr] \\ =&\sum_{i=1}^{\infty}E\bigl[ \vert X \vert ^{q}I\bigl((i-1)^{\alpha}< \vert X \vert \leq i^{\alpha}\bigr)\bigr] \sum_{n=i}^{\infty}n^{\alpha p-1-q\alpha} \\ \leq&C_{1}\sum_{i=1}^{\infty}E \bigl[ \vert X \vert ^{p} \vert X \vert ^{q-p}I \bigl((i-1)^{\alpha}< \vert X \vert \leq i^{\alpha}\bigr) \bigr]i^{\alpha p-q\alpha} \\ \leq& C_{1}E \vert X \vert ^{p}< \infty. \end{aligned}$$

By the proof of (6.3), one has

6.15 $$ H^{**}_{12}\leq\sum_{n=1}^{\infty}n^{\alpha p-1-\alpha}E\bigl[ \vert X \vert I\bigl( \vert X \vert >n^{\alpha}\bigr)\bigr]\leq CE \vert X \vert ^{p}< \infty. $$

Consequently, by (6.10) and (6.12)-(6.15), we obtain $H_{1}<\infty$. So, we obtain the result (3.2). Finally, by the proof of (6.8), (3.3) also holds true. □

### Proof of Theorem 3.3

For some $\beta\geq1$ and $\alpha>(1+\beta)/2$, we take $p=(1+\beta )/\alpha$ and have $\alpha p=1+\beta$. Applying Theorem 3.2, we obtain (3.6) and (3.7) immediately. Combining (3.7) with the Borel-Cantelli lemma, we establish the result of (3.8). □

### Proof of Theorem 3.4

Similar to the proof of Theorem 3.1, by Lemma 2.3, we can check that

6.16 $$ \begin{aligned}[b] &\sum_{n=1}^{\infty}n^{-1-\beta}E\Biggl( \Biggl\vert \sum_{i=1}^{n} A_{ni}X_{i} \Biggr\vert -\varepsilon n^{\alpha} \Biggr)^{+} \\ &\quad \leq C_{1}\sum_{n=1}^{\infty}n^{-1-\beta-\alpha}E\Biggl\vert \sum_{i=1}^{n} \bigl[A_{ni}X_{ni}-E(A_{ni}X_{ni})\bigr] \Biggr\vert ^{2} \\ &\qquad {}+\sum_{n=1}^{\infty}n^{-1-\beta}E \Biggl\vert \sum_{i=1}^{n}A_{ni}\tilde {X}_{ni} \Biggr\vert +\sum_{n=1}^{\infty}n^{-1-\beta} \Biggl\vert \sum_{i=1}^{n}E(A_{ni}X_{ni}) \Biggr\vert \\ &\quad :=Q_{1}+Q_{2}+Q_{3}. \end{aligned} $$

By the proof of (6.3), it follows that

6.17 $$\begin{aligned} Q_{2} \leq&3\sum_{n=1}^{\infty}n^{-1}E\bigl[ \vert X \vert I\bigl( \vert X \vert >n^{\alpha}\bigr)\bigr] =3\sum_{n=1}^{\infty}n^{-1}\sum_{m=n}^{\infty}E\bigl[ \vert X \vert I\bigl(m< \vert X \vert ^{1/\alpha}\leq m+1\bigr)\bigr] \\ =&3\sum_{m=1}^{\infty}E\bigl[ \vert X \vert I\bigl(m< \vert X \vert ^{1/\alpha}\leq m+1\bigr)\bigr]\sum _{n=1}^{m}n^{-1} \\ \leq&C_{1}\sum_{m=1}^{\infty}\log m E \vert X \vert I\bigl(m< \vert X \vert ^{1/\alpha}\leq m+1\bigr)] \leq C_{2}E\bigl[ \vert X \vert \log \vert X \vert \bigr]< \infty . \end{aligned}$$

Similarly, by the proof of (6.4), one has

6.18 $$ Q_{3}\leq C_{1}\sum_{n=1}^{\infty}n^{-1}E\bigl[ \vert X \vert I\bigl( \vert X \vert >n^{\alpha}\bigr)\bigr]\leq C_{2}E\bigl[ \vert X \vert \log \vert X \vert \bigr]< \infty . $$

Moreover, by the proof of (6.5), we obtain

6.19 $$ \begin{aligned}[b] Q_{1} &\leq C_{1}\sum_{n=1}^{\infty}n^{-1-\beta-\alpha}\sum_{i=1}^{n}E(A_{ni}X_{ni})^{2}=C_{1} \sum_{n=1}^{\infty}n^{-1-\beta-\alpha}\sum _{i=1}^{n}EA^{2}_{ni}EX^{2}_{ni} \\ &\leq C_{2}\sum_{n=1}^{\infty}n^{-1-\alpha}E\bigl[X^{2}I\bigl( \vert X \vert \leq n^{\alpha}\bigr)\bigr]+C_{3}\sum_{n=1}^{\infty}n^{\alpha-1} P\bigl( \vert X \vert >n^{\alpha}\bigr) \\ &\leq C_{2}\sum_{i=1}^{\infty}E \bigl[X^{2}I\bigl((i-1)^{\alpha}< \vert X \vert \leq i^{\alpha}\bigr)\bigr] \sum_{n=i}^{\infty}n^{-1-\alpha}+C_{4}E\bigl[ \vert X \vert \log \vert X \vert \bigr] \\ &\leq C_{5}\sum_{i=1}^{\infty}E \bigl[X^{2}I\bigl((i-1)^{\alpha}< \vert X \vert \leq i^{\alpha}\bigr)\bigr]i^{-\alpha}+C_{4}E\bigl[ \vert X \vert \log \vert X \vert \bigr] \\ &\leq C_{6}E \vert X \vert +C_{5}E\bigl[ \vert X \vert \log \vert X \vert \bigr]< \infty. \end{aligned} $$

So, by (6.16)-(6.19), (3.9) holds. In addition, by (3.3) with $p=1$, (3.10) also holds true under the conditions of Theorem 3.4. □

### Proof of Theorem 4.1

It is easy to check that

$$ \frac{1}{n^{r}}\Psi_{n}^{\prime}D_{n}= \begin{bmatrix} \frac{1}{n^{r}}\sum_{i=1}^{n}\alpha_{1}(t_{i}-t_{n})d_{i} \\ \vdots \\ \frac{1}{n^{r}}\sum_{i=1}^{n}\alpha_{m_{0}}(t_{i}-t_{n})d_{i} \end{bmatrix} . $$

In order to prove (4.10), it suffices to look at the *j*th component $\frac{1}{n^{r}}\sum_{i=1}^{n} \alpha_{j}(t_{i}-t_{n})d_{i}$ of $\frac{1}{n^{r}}\Psi_{n}^{\prime}D_{n}$. For some $q>\frac{2\beta}{2r-1}$, by (4.9), we apply Theorem 3.3 with $\alpha=r$, $A_{ni}=\alpha_{j}(t_{i}-t_{n})$ in (4.8) and $X_{n}=d_{n}$, and obtain the result of (4.10).

Moreover, by Assumption 4.1, $W_{0}^{-1}$ exists. In addition, by (4.6) in Assumption 4.2, $(\frac{1}{n^{r}}\Psi_{n}^{\prime}\Psi_{n})^{-1}$ exists and

$$\sigma_{\max}\biggl(\biggl(\frac{1}{n^{r}}\Psi_{n}^{\prime} \Psi_{n}\biggr)^{-1}\biggr)\leq {\frac{1}{\lambda}},\quad \mbox{a.s.}, $$

where $\sigma_{\max}(\cdot)$ is the largest eigenvalue. Therefore, combining

$$ e(t_{n})=W_{o}^{-1}\biggl(\frac{1}{n^{r}} \Psi_{n}^{\prime}\Psi_{n}\biggr)^{-1} \frac {1}{n^{r}}\Psi_{n}^{\prime}D_{n} $$

with (4.10), we obtain (4.11) immediately. □
